# Protein and Peptide in Cancer Research: From Biomarker to Biotherapeutics

**DOI:** 10.3390/cancers17183031

**Published:** 2025-09-17

**Authors:** Joo Hyeong Seo, Seung Hoon Shin, Hye Rin Woo, Yu Rim An, A Hyun Youn, Song Yeon Kim, Mi-Ran Ki, Seung Pil Pack

**Affiliations:** 1 Department of Biotechnology and Bioinformatics, Korea University, Sejong 30019, Republic of Korea; sjh0413@korea.ac.kr (J.H.S.); bimilbunho@korea.ac.kr (S.H.S.); gpfls99017@korea.ac.kr (H.R.W.); ayr7777@korea.ac.kr (Y.R.A.); ahyunannie@korea.ac.kr (A.H.Y.); sorenklin@korea.ac.kr (S.Y.K.); 2 Institute of Industrial Technology, Korea University, Sejong 30019, Republic of Korea; allheart@korea.ac.kr

**Keywords:** protein, peptide, cancer, biomarker, biotherapeutics

## Abstract

Proteins and peptides play a pivotal role in key pathological processes, including cancer growth, immune evasion, angiogenesis, and metastasis. Consequently, they are gaining attention as significant biomolecules in the diagnosis and treatment of various diseases. This review provides a comprehensive overview of the latest research trends and technological advancements in protein- and peptide-based cancer diagnostic and therapeutic strategies. It covers the clinical application of major diagnostic markers such as PSA, CA125, HER2, and AFP, as well as therapeutic strategies including monoclonal antibodies, immune checkpoint inhibitors, and anticancer peptides. Additionally, it introduces quantitative analysis techniques such as ELISA, mass spectrometry, and CyTOF, as well as advancements in delivery systems based on nanoparticle–peptide complexes. Peptides offer advantages for precision therapy due to their high target specificity and structural modifiability. However, they also have limitations such as stability, enzymatic degradation, and delivery efficiency. Emerging technologies such as synthetic biology, computational design, and omics-integrated analysis are being developed to address these challenges, and rapid advancements in this field are enhancing the potential for clinical application. This review underscores the potential of protein- and peptide-based strategies for precision cancer diagnosis and personalized therapy and proposes future research directions in this field.

## Simple Summary

Cancer arises from a complex set of molecular changes that disrupt the normal cell function. Among these, proteins and peptides play central roles in driving tumor development and progression. This research emphasizes the significance of proteins and peptides not only as markers for early cancer detection but also as promising candidates for targeted therapies. Their ability to reflect real-time changes in cellular processes makes them valuable tools for improving diagnostic accuracy and treatment outcomes. Recent technological advancements have enabled more precise identification, characterization, and delivery of protein- and peptide-based agents. By integrating insights from molecular biology, bioengineering, and data science, this field aims to support the development of personalized and more effective cancer treatments. This review discusses the growing effort to enhance cancer care through precision medicine and highlights the expanding impact of protein and peptide research in oncology.

## 1. Introduction

Cancer remains one of the most critical global health challenges, responsible for nearly ten million deaths annually [1]. Its global burden is steadily increasing due to demographic changes, environmental stressors, and lifestyle-related risk factors [2]. Unlike infectious diseases, which typically result from a single causative agent, cancer arises from a complex interplay of genetic mutations, epigenetic dysregulation, and altered cellular signaling pathways [3,4,5]. These disturbances give rise to the hallmarks of cancer, such as uncontrolled proliferation, resistance to apoptosis, sustained angiogenesis, metabolic reprogramming, and immune evasion [6,7].

Molecular regulation of these processes depends on proteins and peptides. Genomic information is converted into structural elements, enzymatic functions, signal transduction, and intercellular communication by proteins. Hormonal regulation, immune modulation, and intercellular signaling involve peptides from larger precursors or signaling molecules. Dysregulated protein expression, mutations, or subcellular mislocalization can drive tumorigenesis, exemplified by the overactivation of receptor tyrosine kinases such as EGFR and HER2, or the loss of tumor suppressors like p53 and PTEN [8,9]. Compared to genomic alterations, proteomic changes more directly reflect the dynamic physiological state of cancer cells and their microenvironment, underscoring the growing importance of proteomics in cancer biology as a functional and clinically relevant layer of molecular information [10,11].

Proteins and peptides are widely used in cancer diagnosis and treatment due to their versatility. Multiple protein-based biomarkers are used for early detection, prognosis, and therapeutic monitoring [12,13]. Representative examples include prostate-specific antigen (PSA) for prostate cancer, cancer antigen 125 (CA125) for ovarian cancer, and HER2 for breast cancer [14]. Among the wide range of protein- and peptide-based biomarkers, several canonical examples have become cornerstones in clinical oncology. Prostate-specific antigen (PSA) remains the most widely used biomarker for prostate cancer, applied in screening, disease monitoring, and therapeutic decision-making. Similarly, human epidermal growth factor receptor 2 (HER2) serves not only as a diagnostic and prognostic marker in breast cancer but also as a predictive biomarker guiding targeted therapies such as trastuzumab and pertuzumab. Cancer antigen 125 (CA125) is routinely applied in ovarian cancer management, particularly for disease monitoring and recurrence detection. These representative cases illustrate how protein biomarkers can serve diverse clinical purposes—ranging from early detection to prognosis and treatment stratification. While their use has transformed cancer management, they also highlight important challenges such as limited specificity, variable sensitivity across populations, and potential over-reliance on single-analyte tests. Therefore, ongoing research increasingly emphasizes multiplexed assays, proteomic signatures, and integrative biomarker panels that move beyond reliance on individual markers like PSA, HER2, or CA125 to capture the complex biology of cancer more comprehensively. Blood sampling or biopsy can detect these secreted or membrane-bound markers. Mass spectrometry, multiplex ELISAs, and immunoaffinity enrichment have accelerated the discovery and validation of novel protein and peptide biomarkers [15,16,17].

Beyond diagnosis, proteins and peptides have become pivotal in treatment strategies. Monoclonal antibodies (mAbs) are one of the most successful classes of biologics, functioning either as receptor blockers (e.g., cetuximab) or as carriers in antibody–drug conjugates (ADCs) that deliver cytotoxic agents specifically to tumor cells [18,19,20]. In parallel, peptides are being developed as targeted therapeutics due to their favorable pharmacokinetics, rapid tissue penetration, synthetic flexibility, and tunable binding specificity [21,22,23]. Therapeutic peptides include receptor-specific ligands, tumor-penetrating sequences, and pro-apoptotic agents designed to maximize efficacy and minimize systemic toxicity [24,25]. Moreover, proteomic profiling of extracellular vesicles and exosomes is emerging as a non-invasive liquid biopsy strategy for real-time monitoring of tumor evolution [26,27].

Recent advances in drug design and delivery have significantly expanded the clinical potential of protein- and peptide-based therapeutics. Unlike traditional small-molecule drugs, these biologics offer improved target specificity, reduced adverse effects, and enhanced pharmacodynamic profiles, making them increasingly attractive in clinical settings where precision and safety are paramount [28].

A major driving force behind this progress is the integration of artificial intelligence and machine learning into the drug development process. These computational tools have revolutionized how researchers predict molecular interactions, assess structural stability, and model in vivo performance [29,30]. By streamlining these critical steps, AI and ML have accelerated the discovery and optimization of protein and peptide drugs, enabling more efficient and targeted therapeutic design.

In parallel, large-scale molecular profiling of cancer tissues using advanced proteomic and peptidomic technologies has deepened our understanding of disease biology. These approaches have highlighted the importance of post-translational modifications, protein–protein interactions, and short regulatory peptides in cancer progression and therapeutic resistance [31,32].

Nanomedicine has introduced sophisticated platforms—such as liposomes, polymeric carriers, and inorganic nanoparticles—that protect therapeutic proteins and peptides from degradation, extend their circulation time, and enhance their accumulation in tumor tissues [33,34,35]. These delivery vehicles not only improve the pharmacokinetics and efficacy of biologics but also support real-time imaging capabilities [36,37,38]. This convergence of targeted delivery and diagnostic imaging has given rise to theragnostic strategies, which combine therapy and diagnosis within a single platform.

Advances in synthetic biology, systems medicine, and multi-omics integration will accelerate the application of proteins and peptides in cancer therapy. Rational design and modular engineering are improving the stability, immunocompatibility, and multifunctionality of new protein scaffolds and synthetic peptides [39,40]. This includes the development of peptide–drug conjugates, bioswitchable therapeutics, and *de novo* protein constructs that can respond dynamically to tumor microenvironmental cues.

Peptide- or antibody-based biosensor-based point-of-care diagnostic technologies are being optimized for use in various medical settings, including those with limited infrastructure [41,42]. Additionally, the integration of proteomics data with genomic, epigenomic, and metabolomic data provides unprecedented insights into tumor biology characterization.

Spatial proteomics and single-cell proteogenomics are revealing the microstructural organization of tumor cells and their interactions with the stroma and immune cells [43]. These findings contribute to explaining tumor heterogeneity and identifying new therapeutic vulnerabilities.

Technological and conceptual advancements are revolutionizing the function of proteins and peptides in cancer research. These biomolecules represent the spirit of precision cancer care as diagnostic, monitoring, and therapeutic platforms, and they are expected to lead the way in cancer research in the future.

Distinct from previous reviews, which have often addressed diagnostic biomarkers or therapeutic peptides and proteins in isolation, this article provides an integrative framework that captures the full translational continuum—from molecular mechanisms to clinical applications. By incorporating recent innovations such as artificial intelligence, nanomedicine, multi-omics integration, spatial proteomics, and synthetic biology, we highlight how proteins and peptides are being repositioned as central pillars of precision oncology. This holistic approach not only synthesizes current knowledge but also identifies emerging directions, thereby offering a unique and forward-looking perspective that fills an important gap in the literature.

Given these rapidly advancing clinical and technological developments, there is a clear need for an updated, integrative review that comprehensively summarizes the evolving role of proteins and peptides in oncology. While previous reviews have focused separately on either diagnostic or therapeutic applications, or specific protein types, few have captured the full translational continuum from molecular mechanisms to clinical implementation within the framework of modern precision medicine.

This review aims to bridge that gap by providing a cohesive overview of recent progress in the understanding, application, and technological enhancement of proteins and peptides in cancer. We examine their roles in tumor biology, their utility as biomarkers and therapeutic agents, and the impact of emerging innovations such as omics integration, machine learning, and nanotechnology. Through this synthesis, we highlight how proteins and peptides are no longer peripheral components but are increasingly becoming central to the diagnosis, treatment, and long-term management of cancer. Finally, we propose future directions for this field.

## 2. Proteins and Peptides as Biomarkers in Cancer

Cancer remains a leading cause of death globally, largely due to the lack of reliable early diagnostic tools and the difficulty in predicting treatment outcomes. These challenges are compounded by the histological and molecular heterogeneity of cancer—variations in cellular structure, tissue architecture, and molecular profiles that differ not only between cancer types but also within individual tumors. This complexity undermines the accuracy of conventional diagnostic methods and complicates therapeutic decision-making [44,45].

To address these limitations, researchers are increasingly focused on identifying biomarkers—biological indicators that reflect specific disease states. Among the various biomolecule classes, proteins and peptides stand out as promising candidates due to their functional diversity, detectability in non-invasive biofluids like blood and urine, and relative ease of analysis. Proteins such as enzymes, receptors, and cytokines are often overexpressed or secreted by tumor cells or the surrounding microenvironment. Peptides, typically short amino acid sequences resulting from degradation or abnormal translation, are sensitive to acute physiological changes and useful for real-time monitoring [46,47].

The ways proteins are released include packaging into extracellular vesicles (EVs), active secretion through secretory vesicles, or leakage during cell death processes such as necrosis or apoptosis. EVs are tiny bubbles made of fat that come from cells and carry proteins, RNA, and lipids, helping cells communicate with each other; researchers are now looking into using them to transport biomarkers [48].

Once they are released, proteins usually remain stable because of disulfide bonds and their correct shape, and they can also resist being broken down by enzymes, which helps them stay in the bloodstream for a longer time [49]. In addition to disulfide bonding, secondary structural elements such as α-helices and β-sheets play a critical role in maintaining conformational stability. These motifs enhance resistance to proteolysis and stabilize domains that often serve as biomarker epitopes. Moreover, higher-order conformational features influence antigen–antibody recognition, directly affecting the sensitivity and specificity of biomarker detection assays. Many of these proteins are selectively expressed in specific cancer types or molecular subtypes, contributing to their disease specificity and utility in diagnosis and prognosis [50].

In contrast, peptides are generally composed of short amino acid sequences derived from proteolytic degradation or aberrant translation. Due to their structural instability, they exhibit a short half-life in circulation [51], yet they are highly sensitive to acute physiological changes such as inflammation, stress, or metabolic fluctuations [52]. Peptides offer distinct advantages as diagnostic and therapeutic agents due to their favorable properties: minimal immunogenicity, high tissue penetration, ease of chemical modification, small size, and inherent specificity and flexibility in targeting cancer cells while minimizing harm to healthy tissues [53,54]. These characteristics make peptides suitable targets for biosensors designed to monitor real-time physiological responses, and they are particularly valuable in early diagnosis and treatment monitoring [54].

### 2.1. Protein Biomarkers: Concepts and Clinical Applications

Clinically validated protein-based biomarkers such as human epidermal growth factor receptor 2 (HER2), prostate-specific antigen (PSA), cancer antigen 125 (CA125), carcinoembryonic antigen (CEA), and alpha-fetoprotein (AFP) are already widely used for early detection, prognosis, and monitoring of therapeutic efficacy across various cancer types (Table 1) [50]. HER2 is a critical prognostic and predictive marker for breast cancer, and its overexpression is strongly associated with increased tumor aggressiveness, poorer clinical outcomes, and resistance to certain therapies, including hormonal therapy and the CMF (cyclophosphamide, methotrexate, and fluorouracil) chemotherapy regimen [55]. Conversely, targeted therapies such as trastuzumab significantly improve survival in patients with HER2-positive breast cancer [47]. Beyond HER2, estrogen receptors (ER) and progesterone receptors (PR) are crucial prognostic and predictive markers for breast cancer. PR loss has been associated with endocrine resistance and poorer prognosis [56].

PSA is a protein produced by normal prostate cells, and a sharp rise in PSA levels in the blood may indicate prostate cancer [55]. PSA testing plays an important role in evaluating treatment response, monitoring tumor progression, and identifying men who may need a prostate biopsy [57]. CA125 is commonly observed in epithelial ovarian cancer and various other gynecological and non-gynecological malignant tumors. Although widely used, CA125 has low sensitivity in the early stages of ovarian cancer and may also be elevated in benign conditions such as menstruation or endometriosis [55]. Concurrent evaluation with human epididymis protein 4 (HE4) yields more optimal diagnostic results [58].

CEA is a serum marker primarily used for monitoring colorectal cancer, assessing prognosis, post-surgical follow-up, and tracking disease progression. It is also tested in other cancers, including breast cancer, liver cancer, ovarian cancer, pancreatic cancer, and prostate cancer [55]. A major limitation of CEA is its low diagnostic specificity, as it can be expressed in non-tumor tissues, leading to potential misinterpretation [50]. AFP serves as a key diagnostic marker for hepatocellular carcinoma [46]. It is also used in detecting hepatoblastoma, certain tumors of the ovary and testis, and malignancies of the gastrointestinal tract [55]. Carbohydrate antigen 19-9 (CA19-9), a major marker for pancreatic ductal adenocarcinoma, is also elevated in colorectal cancer, other gastrointestinal malignant tumors, and cholangiocarcinoma [55]. Adenocarcinomas such as gastric, colorectal, breast, and lung cancers exhibit high levels of carbohydrate antigen 72-4 (CA72-4), a blood tumor marker used to diagnose, monitor, and prognose gastric cancer [55].

p53 plays a crucial role in cell cycle regulation and DNA damage response. Cancer cells can bypass normal cell division and grow endlessly due to p53 gene mutations, one of the most prevalent genetic changes in human cancer [59]. p53 mutations are predictive factors in lung, colorectal, and pancreatic cancers and can cause chemotherapy resistance, necessitating alternative treatments [55]. Lactate dehydrogenase (LDH) catalyzes the reversible transfer of pyruvate to lactate, and high serum LDH levels are linked to poor survival in solid tumors, serving as a valuable and affordable prognostic biomarker [60]. Neuron-specific enolase (NSE), enriched in neurons and peripheral neuroendocrine cells, is a clinically relevant biomarker for staging, monitoring treatment, and predicting relapse in small cell lung cancer (SCLC) [60].

Other protein biomarkers include Ki-67, anaplastic lymphoma kinase (ALK), ROS proto-oncogene 1 (ROS1), DNA mismatch repair (MMR) proteins, neurotrophic receptor tyrosine kinases (NTRK), and programmed death-ligand 1 (PD-L1), all of which are found in various solid tumors [50].

### 2.2. Peptide Biomarkers: Concepts and Clinical Applications

Peptide biomarkers constitute a distinct class of cancer indicators with unique diagnostic and therapeutic potential. Unlike proteins, they are often short-lived in circulation due to structural instability, yet their favorable properties—including minimal immunogenicity, high tissue penetration, and ease of chemical modification—enable selective tumor targeting and real-time physiological monitoring [53,54].

Representative peptide biomarkers include pro-brain natriuretic peptide (pro-BNP), which has been applied in cardio-oncology to monitor chemotherapy-related cardiac dysfunction, although it lacks cancer specificity [54]. Hepcidin, associated with iron metabolism, has been investigated as a biomarker in hepatocellular carcinoma, but its levels can be confounded by inflammation [55]. Chromogranin A serves as a diagnostic and monitoring marker for neuroendocrine tumors, though false positives may occur in renal failure [56]. Thymosin β4 has been linked to invasion and metastasis in breast and colon cancers, but its clinical validation remains limited [57].

In addition to these clinically investigated peptides, research has identified non-coding RNA-derived micropeptides, gastrin-releasing peptide (GRP) and its precursor pro-GRP, and tumor-homing peptides such as iRGD, angiopep2, and PL3, which target tumor-specific receptors and microenvironment components. The therapeutic and diagnostic potential of peptides in oncology is summarized in Table 2.

The identification of novel serum peptide biomarkers for early and late recurrence in cholangiocarcinoma (CCA) underscores the significant potential of peptidome approaches to enhance accuracy in classifying cancer recurrence [53]. Matrix-Assisted Laser Desorption/Ionization with Time-of-Flight Mass Spectrometry (MALDI-TOF MS) has proven effective in differentiating cancer recurrence types based on specific mass signatures, identifying distinct peptide mass fingerprints (PMFs) for early versus late recurrence [53]. KNTC1, DCLK1, ALG10, ATR, POLA1, BLM, SP100, and PPP1R15A were identified as being upregulated in early CCA recurrence. These peptides are implicated in critical cellular processes such as DNA stress response and the maintenance of genomic instability. Conversely, for late recurrence, peptides like SERPINA1, TGFB2, SERPING1, and CAD were identified, with roles in immunosuppression, epithelial-to-mesenchymal transition (EMT), and pyrimidine synthesis [53].

Non-coding RNA-derived micropeptides (MPs) represent a complex class of biomarkers and potential therapeutics, derived from non-coding RNAs. Research is actively exploring their expression patterns in breast cancer subtypes, identifying micropeptides that are differentially expressed in tumor versus non-tumor samples and those exhibiting tumor or subtype specificity [61]. These micropeptides are predicted to influence the tumor immune environment and participate in key cellular processes such as cell communication, metabolism, and signaling [61].

Gastrin-releasing peptide (GRP) is a gastrointestinal hormone present in normal bronchial epithelial cells, pulmonary fibroblasts, central nervous system cells, and neuroendocrine cells. Due to GRP’s short half-life and instability, it is more suitable to detect its precursor, pro-GRP. The concurrent use of pro-GRP and NSE enhances the sensitivity of SCLC detection [60].

Phage display technology is a powerful and versatile platform for the discovery of novel peptide biomarkers, particularly for early cancer detection [54]. These peptides are generally oligopeptides, ranging from 5 to approximately 30 amino acids in length [62]. Tumor-homing peptides are incorporated into specific target cells via endocytosis, binding with affinity to specific cell surface molecules such as receptors and receptor-associated proteins that are overexpressed in tumor or tumor microenvironment cells [62]. Tumor-targeted peptides identified through phage display include PL3, angiopep2, and iRGD. PL3 targets Tenascin-C and is absorbed through endocytosis. Angiopep2 targets LRP1 and is absorbed through endocytosis. iRGD targets αvβ3 integrin and Neuropilin-1 (NRP-1), and is absorbed through endocytosis after degradation [62]. The CV-1 peptide, which specifically binds to CD44 (involved in tumor progression and drug resistance), is a promising probe for early molecular imaging of CD44v-positive gastric tumors [53]. A plectin-1 targeted peptide (PTP) that binds to plectin-1, a marker of pancreatic ductal adenocarcinoma (PDAC), has been applied as a targeted imaging agent. A novel peptide with high specificity and sensitivity toward hepsin, an emerging marker of prostate cancer, has been applied to improve pharmacokinetics and enhance cancer detection [53].

Reliance on a single biomarker often leads to limited sensitivity and specificity, resulting in false positives or negatives that undermine diagnostic accuracy and clinical reliability [60]. To address this limitation, multiplex biomarker panels—combining multiple molecular indicators—are being developed to produce integrated diagnostic signatures. This strategy relies on high-dimensional molecular data derived from omics technologies such as proteomics, transcriptomics, and metabolomics, which are routinely used to analyze patient samples. A multiomic biomarker panel, which combines different factors found in patients’ blood and the fluid from their pancreatic cysts, has proven to be very accurate in telling apart patients who are at low risk from those at high risk for developing pancreatic cancer. For pancreatic ductal adenocarcinoma (PDAC), studies have identified multi-biomarker combinations that achieve high specificity and sensitivity, notably outperforming single markers like CA19-9 and CEA in diagnostic performance [53,63].

When paired with machine learning algorithms, these data sets enable the construction of cancer-specific diagnostic tools, prognostic models, and predictors of treatment response (Table 3). For instance, a random forest model trained using data from “The Cancer Genome Atlas (TCGA)” has successfully predicted overall survival outcomes in breast cancer [64]. Similarly, protein panels based on multiple reaction monitoring (MRM) have differentiated hepatocellular carcinoma patients from healthy individuals [65]. For example, a random forest model trained on data from TCGA has demonstrated the ability to predict overall survival in breast cancer patients, while multiplexed MRM-based protein panels have successfully differentiated hepatocellular carcinoma patients from healthy controls [66]. Among novel biomarkers, ORF1p originating from LINE-1 retrotransposons has demonstrated promise for early ovarian cancer detection. Its inclusion in multi-analyte panels improves diagnostic precision, aids in monitoring therapeutic responses in gastroesophageal cancers, and serves as a prognostic indicator for overall survival in both gastroesophageal and colorectal cancers [67].

Advancements in multi-omics integration platforms have further propelled the discovery of innovative biomarkers [72]. By analyzing interconnected biological data across genomic, transcriptomic, proteomic, and metabolomic levels, researchers aim to capture the complexity of human physiology [72]. Machine learning algorithms are central to this effort, enabling the identification of clinically relevant biomarkers and the development of highly accurate predictive models [73].

To be considered clinically useful, protein and peptide biomarkers must fulfill a set of rigorous standards that ensure both their diagnostic and prognostic value [74,75,76]. Firstly, these biomarkers should exhibit differential expression patterns that are specific to distinct cancer types or subtypes [12]. This characteristic enables target detection and supports the accurate classification of malignancies. Equally important is the ability to detect these biomarkers reliably and reproducibly in biofluids such as blood, urine, or saliva [77]. Consistent detection across samples and settings enhances clinical confidence and facilitates non-invasive diagnostic procedures. Furthermore, there must be a statistically significant correlation between the biomarker’s presence or expression levels and clinically relevant parameters—such as disease progression, therapeutic response, or overall prognosis [59]. This relationship allows clinicians to make informed decisions throughout a patient’s treatment journey. Lastly, biomarkers must be quantifiable in clinical environments with high sensitivity and specificity [78]. The capability to measure them precisely underpins their utility in real-world diagnostic workflows, ensuring that both false positives and false negatives are minimized.

### 2.3. Methodologies and Quantitative Analytical Technologies for Protein and Peptide Biomarker Discovery

To provide a deeper understanding of how protein and peptide biomarkers are identified, validated, and applied in oncology, it is essential to outline both the methodologies that underpin their discovery and the technologies that enable their precise quantification. Together, these approaches provide a comprehensive framework for translating candidate biomarkers into clinically useful diagnostic and prognostic tools.

The discovery of protein and peptide biomarkers relies on a wide range of experimental and computational approaches that enable the identification, validation, and clinical translation of candidates. These methodologies have significantly advanced our ability to improve diagnostic accuracy, therapeutic monitoring, and prognostic assessment in cancer.

Mass spectrometry-based approaches such as Matrix-Assisted Laser Desorption/Ionization with Time-of-Flight Mass Spectrometry (MALDI-TOF MS) have proven effective in distinguishing cancer recurrence types based on specific mass signatures. For example, distinct peptide mass fingerprints (PMFs) were identified for early versus late recurrence in cholangiocarcinoma (CCA), where peptides such as KNTC1, DCLK1, ALG10, ATR, and BLM were associated with early recurrence, while SERPINA1, TGFB2, and SERPING1 were implicated in late recurrence [53].

Non-coding RNA-derived micropeptides (MPs) represent another complex class of biomarkers with potential therapeutic relevance. These micropeptides, derived from non-coding RNAs, exhibit tumor- or subtype-specific expression patterns and influence key cellular processes such as immune regulation, metabolism, and cell signaling [61]. Similarly, peptide hormones like gastrin-releasing peptide (GRP) and its more stable precursor pro-GRP, when combined with established markers such as NSE, improve sensitivity in detecting small cell lung cancer (SCLC) [60].

Phage display technology provides a versatile platform for the discovery of tumor-homing and tumor-targeted peptides. These oligopeptides (5–30 amino acids) are identified through affinity binding to overexpressed receptors or tumor microenvironment proteins. Examples include PL3 (Tenascin-C), angiopep2 (LRP1), and iRGD (αvβ3 integrin and Neuropilin-1), all of which exhibit tumor-selective uptake via endocytosis [62]. Additional examples are the CV-1 peptide targeting CD44 in gastric tumors, plectin-1 targeted peptides for pancreatic ductal adenocarcinoma (PDAC), and hepsin-specific peptides for prostate cancer imaging and detection [53].

Multiplex and multi-omics biomarker panels have been developed to overcome the limitations of single biomarkers, which often lack sufficient sensitivity and specificity. Multi-analyte signatures derived from proteomics, transcriptomics, and metabolomics have shown improved accuracy, such as in differentiating low-risk versus high-risk pancreatic cancer patients. In PDAC, panels combining multiple biomarkers outperformed single markers like CA19-9 and CEA in diagnostic performance [63].

Machine learning approaches are increasingly integrated with multi-omics datasets to construct cancer-specific diagnostic tools, prognostic models, and predictors of therapeutic response. For example, a random forest model trained on The Cancer Genome Atlas (TCGA) data accurately predicted overall survival in breast cancer [64]. Similarly, multiplexed protein panels analyzed through multiple reaction monitoring (MRM) distinguished hepatocellular carcinoma patients from healthy individuals [65,66]. Novel biomarkers such as ORF1p from LINE-1 retrotransposons also illustrate how machine learning-assisted integration enhances precision, improves therapeutic monitoring, and supports prognostic evaluation in multiple cancers [67].

Principles of machine learning for biomarker discovery involve supervised and unsupervised algorithms that can process high-dimensional omics data. Supervised methods, such as random forest or support vector machines, are trained on labeled datasets to classify patients or predict outcomes. Unsupervised methods, such as clustering, identify hidden patterns within biomarker data without prior labels. These algorithms facilitate feature selection, identify clinically relevant signatures, and construct predictive models with high sensitivity and specificity [73].

To be clinically useful, biomarkers identified through these methodologies must meet rigorous criteria. They should demonstrate cancer type-specific expression patterns, reproducibility across biofluids such as blood or urine, and statistically significant correlations with disease progression, therapeutic response, or prognosis [74,75,76,77]. Furthermore, biomarkers must be quantifiable in clinical workflows with high sensitivity and specificity to minimize false positives and negatives, ensuring their translational value in real-world diagnostics [78].

### 2.4. Quantitative Analytical Technologies: ELISA, Mass Spectrometry, and Emerging High-Sensitivity Platforms

The clinical implementation of protein and peptide biomarkers depends heavily on quantitative analytical technologies that ensure precise and reproducible measurements. Unlike qualitative or semi-quantitative approaches, these methods allow accurate numerical quantification of target molecules, thereby significantly enhancing the reliability of biological interpretations. In oncology, quantitative precision is essential, as biomarker concentrations are closely linked to decisions regarding treatment timing, drug response prediction, and disease staging.

Quantitative analytical methods refer to experimental techniques that detect specific proteins or peptides in biological samples and express their abundance in measurable units. These approaches serve as critical tools to improve diagnostic sensitivity and specificity, providing objective data for assessing disease progression, therapeutic response, and patient prognosis. The most widely adopted technologies in both clinical and research settings are as follows (Table 4).

First, enzyme-linked immunosorbent assay (ELISA) quantifies target proteins by employing the binding between antigens and antibodies, coupled with enzyme-mediated signal detection. Known for its high sensitivity and specificity, ELISA is widely used in hospitals and diagnostic laboratories. It has been applied to the clinical quantification of tumor markers such as HER2 and CEA. However, its single-analyte design limits its scalability for multiplexed analysis [79,80].

Second, multiple reaction monitoring (MRM) is a targeted mass spectrometry approach that uses a triple quadrupole instrument to selectively monitor specific mass-to-charge (*m*/*z*) transitions of peptides. This method enables simultaneous quantification of multiple proteins or peptides with high sensitivity and specificity. MRM has been applied to quantify a panel of biomarkers, such as GP73, AFP, and DKK1, in the plasma of hepatocellular carcinoma patients, demonstrating its utility in multiplex profiling. Nonetheless, its dependence on high-end instrumentation and technical expertise remains a limiting factor [82,83].

Third, the proximity extension assay (PEA) involves DNA-tagged antibody pairs that bind to the same protein target. When in proximity, a DNA polymerase-mediated extension generates amplicons that are subsequently quantified by qPCR or next-generation sequencing. PEA is highly sensitive and allows for multiplexed analysis from minimal sample volumes, making it well suited for clinical diagnostics. For example, PEA has been used to simultaneously quantify 92 proteins from the plasma of ovarian cancer patients, aiding early diagnosis and staging efforts [84,85].

Fourth, cytometry by time of flight (CyTOF) uses metal isotope-labeled antibodies and time-of-flight mass spectrometry to quantify dozens of proteins at single-cell resolution. Unlike fluorescence-based flow cytometry, CyTOF avoids spectral overlaps and enables high-dimensional phenotyping. This technology has been employed to characterize immune cell subsets, infer cell-to-cell interactions within the tumor microenvironment, and predict responses to immunotherapy. Studies have shown that CyTOF can effectively identify immune cell subsets associated with responsiveness to immune checkpoint inhibitors [86].

These technologies vary in terms of sensitivity, multiplexing capacity, and ease of standardization. For instance, ELISA offers strong clinical utility but is limited in multiplexing. MRM supports simultaneous multi-analyte detection but is costly [87]. PEA and CyTOF represent ultra-sensitive platforms and are gaining attention as next-generation clinical tools [86,88].

Emerging technologies are expected to further enhance the sensitivity and applicability of biomarker quantification. Digital ELISA, based on microfluidic platforms and nanoparticle-enhanced detection systems, is being developed to achieve femtogram-level detection in clinical settings. For example, single-molecule array (Simoa) technology offers more than a thousand-fold increase in sensitivity compared to traditional ELISA, making it ideal for early biomarker detection [89]. In parallel, gold nanoparticle-based plasmonic sensing is being explored for its utility in probing protein–protein interactions and detecting ultra-low abundance biomarkers [90,91,92,93].

### 2.5. Case Studies of Biomarker Discovery Using Omics-Based Approaches

The advancement of precision-oriented quantitative analytical technologies has significantly expanded the clinical applicability of omics-based biomarker discovery. An integrative analysis of breast cancer patient chores using data from The Cancer Genome Atlas (TCGA) and the Clinical Proteomic Tumor Analysis Consortium (CPTAC) identified several genes, including GRB7, INPP4B, and MLPH, that are specifically overexpressed in the HER2-positive subtype [68]. GRB7 is an adaptor protein involved in growth signal transduction and cell motility, and its overexpression has been associated with poor prognosis [69]. INPP4B functions as a regulator of the PI3K-AKT signaling pathway and exhibits tumor suppressive or oncogenic activity in a cancer type-specific manner. MLPH is a protein implicated in melanosome transport, with expression changes observed in HER2-positive tumors, although its mechanistic role remains to be elucidated. These molecules have been proposed as auxiliary biomarkers to complement HER2 expression in evaluating therapeutic response and clinical outcomes.

In another example, a multi-omics analysis of ovarian cancer tissue and plasma samples identified novel candidate proteins that may overcome the limitations of the conventional biomarker CA125. Specifically, WFDC2 (also known as HE4) and SLPI were found to be differentially expressed at both transcriptomic and proteomic levels and have demonstrated significant predictive power in early diagnosis and disease staging [70]. These biomarkers, when combined with CA125, contribute to multiplex diagnostic panels that enable more accurate and refined clinical assessment [71].

Taken together, omics-based quantitative diagnostic strategies facilitate an integrative approach that transcends the limitations of single biomarkers. This paradigm is advancing the realization of precision medicine and is expected to provide a robust foundation for prognosis prediction and personalized therapeutic planning across a range of cancer types.

## 3. Roles of Proteins and Peptides in Tumor Biology

Tumorigenesis is driven by the dysregulation of a complex network of intracellular and extracellular signaling pathways, including selective growth and proliferative advantage, control of stress response, vascularization, and immune modulation [94]. Central to this process are proteins and peptides that function as key signaling molecules, structural mediators, or modulators of the tumor microenvironment [95]. Their aberrant expression or activity alters normal cellular homeostasis and contributes to each stage of cancer development and progression [96].

Well-known signaling pathways involved in cell growth, differentiation, and survival include the mitogen-activated protein kinase (MAPK)/extracellular signal-regulated kinase (ERK) pathway. Mutations in upstream regulators such as RAS and BRAF, associated with this signaling, result in the indefinite proliferation of cancer cells [97]. The PI3K/AKT/mTOR pathway, which regulates cell survival, metabolism, and protein synthesis, is commonly activated in cancers via mutations or amplification of PI3K subunits, PTEN loss, or growth factor receptor overexpression [98]. The Wnt/β-catenin pathway, involved in cell fate specification and stem cell maintenance, also becomes oncogenic when β-catenin escapes degradation and accumulates in the nucleus, where it induces transcription of pro-tumorigenic genes [99]. The Notch signaling pathway, known to control differentiation, proliferation, apoptosis, adhesion, and epithelial-to-mesenchymal transition (EMT), contributes to tumor heterogeneity and plasticity by maintaining cancer stem-like cell populations when aberrantly activated [100]. Lastly, the p53 protein acts as a genomic gatekeeper by sensing DNA damage and activating genes involved in cell cycle arrest, DNA repair, and apoptosis [101].

**Figure 1:**
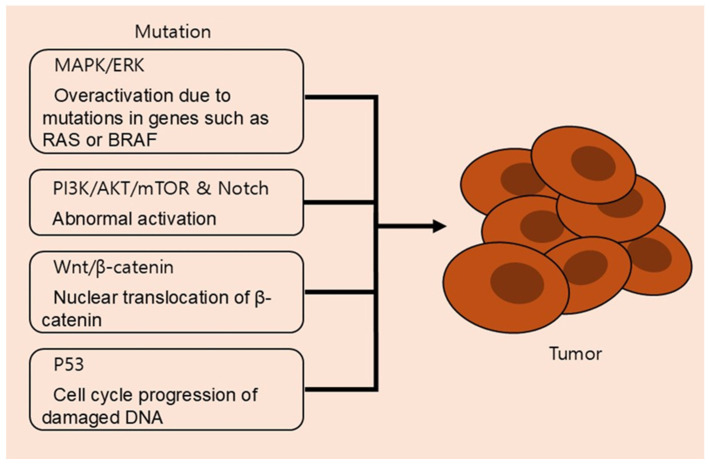
Key signaling pathway mutations driving tumor development.

Figure 1 illustrates how mutations in the following common signaling pathways contribute to tumor formation. In the MAPK/ERK pathway, if a mutation occurs in key regulatory genes like RAS or BRAF, cells enter a state of infinite proliferation even without external signals. The PI3K/AKT/mTOR pathway promotes the survival and growth of cancer cells when the pathway is activated due to mutations in the PI3K gene or the loss of the PTEN gene. In the Wnt/β-catenin pathway, the accumulation of β-catenin in the nucleus induces the transcription of tumor-promoting genes. Abnormal activation of the Notch pathway can also contribute to cancer treatment resistance and recurrence by maintaining cancer stem cells.

Deregulation of apoptotic signaling pathways enables tumor cells to evade programmed cell death despite oncogenic stress and genotoxic insults [102]. The intrinsic mitochondrial pathway, tightly controlled by Bcl-2 family proteins, is frequently altered in cancer. Overexpression of Bcl-2 impairs mitochondrial apoptosis by sequestering pro-apoptotic BH3-only proteins (e.g., Bax, Bak), preventing mitochondrial outer membrane permeabilization and the release of cytochrome c [103]. In addition, Bcl-2 interacts with the inositol 1,4,5-trisphosphate receptor (IP3R) on the endoplasmic reticulum to regulate Ca^2+^ flux, further modulating mitochondrial function and apoptosis sensitivity [104]. By binding to Beclin-1, Bcl-2 also suppresses autophagy initiation, thereby facilitating the accumulation of damaged organelles and enhancing cell survival under stress conditions [105].

Resistance to apoptosis is further compounded by mutations in tumor suppressor genes such as TP53 [106]. However, loss-of-function mutations or deletion of TP53 interfere with DNA binding and prevent the expression of p53 target genes. This not only disrupts these protective responses but also confers selective growth advantages to tumor cells [107]. In certain cases, mutant p53 gains novel oncogenic functions, such as promoting metastasis or altering metabolic flux, thereby accelerating tumor progression [108]. In this context, therapeutic efforts have focused on restoring wild-type p53 activity or destabilizing mutant forms using small molecules and peptides.

In addition to regulating the apoptosis signaling pathway, abnormalities in proteins involved in DNA damage recognition and repair are also important determinants of tumorigenesis. When DNA damage occurs, p53 is phosphorylated by ATR, ATM, and CHK1/2, which leads to cell cycle arrest, activation of DNA damage response (DDR) genes, or apoptosis. During this process, key regulators of the DDR, such as ATR, ATM, CHK1/2, and BRCA1/2, are frequently mutated or inactivated in cancer, resulting in genomic instability and an increased mutational burden [108]. For example, loss of BRCA1 or BRCA2 function impairs homologous recombination repair of double-strand breaks, rendering cells reliant on error-prone pathways and susceptible to further genomic insults [109]. This vulnerability underlies the synthetic lethality exploited by PARP inhibitors in BRCA-mutated tumors [110]. Importantly, emerging peptide-based approaches are being developed to mimic or inhibit DDR proteins, with the aim of sensitizing tumor cells to DNA-damaging agents or blocking compensatory repair mechanisms [111].

During tumor growth and progression, specific proteins are secreted into the tumor microenvironment (TME), where they modulate intercellular communication, extracellular matrix (ECM) remodeling, and angiogenesis. Epidermal growth factor (EGF) and vascular endothelial growth factor (VEGF) are canonical examples that orchestrate autocrine and paracrine signaling to support proliferation and neovascularization [112]. Specifically, epidermal growth factor (EGF) is recognized by its receptor, epidermal growth factor receptor (EGFR), and exerts diverse cellular effects. Upon ligand binding, EGFR leads to the activation of adaptor proteins such as growth factor receptor-bound protein 2 (GRB2) and Src homology and collagen (SHC). GRB2 subsequently recruits the guanine nucleotide exchange factor Son of Sevenless (SOS) to the plasma membrane, where it facilitates the exchange of GDP for GTP on RAS, thereby promoting RAS activation, while SHC enhances the efficiency of RAS activation by up to 500-fold. Activated RAS then recruits RAF to the membrane, initiating a phosphorylation cascade that sequentially activates mitogen-activated protein kinase kinase (MEK) and extracellular signal-regulated kinase (ERK). The phosphorylated ERK translocates into the nucleus, where it activates transcription factors including c-FOS, c-MYC, and ETS Like-1 protein (ELK-1) [113].

EGFR also modulates the PI3K-AKT-mTOR pathway. Activation of PI3K results in the conversion of phosphatidylinositol (4,5)-bisphosphate (PIP2) into phosphatidylinositol (3,4,5)-trisphosphate (PIP3), which serves as a docking site for AKT. Recruitment of AKT to the membrane facilitates its phosphorylation by phosphoinositide-dependent kinase-1 (PDK1) and the mTOR complex 2 (mTORC2), leading to downstream effects on cell proliferation and survival. Moreover, activated AKT phosphorylates tuberous sclerosis complex 2 (TSC2), thereby activating mTOR complex 1 (mTORC1), which regulates cell growth, inhibits autophagy, and promotes the expression of growth factors [114].

VEGF-A becomes phosphorylated upon binding to vascular endothelial growth factor receptor-2 (VEGFR-2) and specifically binds to PLCγ. This subsequently activates the PKCβ pathway within the PKC family, contributing to vascular endothelial cell proliferation and angiogenesis through MAPK activation [115]. It also facilitates cell migration and survival via the PI3K/AKT pathway [116]. VEGF and EGF can function synergistically, as VEGF expression is often upregulated by EGFR-mediated signaling and vice versa, creating a feed-forward loop that enhances tumor vascularization [117].

Even after tumor growth, various factors are involved in tumor survival. Transforming growth factor-β (TGF-β) represents a context-dependent cytokine with tumor-suppressive functions in early carcinogenesis and pro-tumorigenic effects in later stages. In advanced tumors, TGF-β promotes epithelial–mesenchymal transition (EMT), immunosuppression, and ECM remodeling. In T cells, TGF-β transcriptionally represses genes encoding several key proteins, such as perforin, granzyme, and IFN-γ, thereby negatively regulating the activity of cytotoxic T lymphocytes (CTLs), which destroy tumor cells. In addition, TGF-β inhibits the expression of NKp30 and NKG2D receptors, which not only suppresses the activity of cytolytic natural killer (NK) cells but also reduces IFN-γ secretion due to NK cell accumulation [108].

Beyond immune modulation, TGF-β also supports the maintenance of cancer stem cell (CSC) phenotypes [118]. CSCs are a subpopulation of tumor cells with enhanced self-renewal, differentiation potential, and resistance to therapy [119]. Proteins such as Nanog, Sox2, and Oct4, commonly associated with embryonic stem cells, are aberrantly expressed in various cancers and sustain the tumor-initiating capacity of CSCs [120]. These transcription factors interact with core signaling networks, including Wnt, Notch, and Hedgehog, forming a regulatory circuit that underpins tumor plasticity, heterogeneity, and relapse after therapy [121]. Disruption of CSC-associated proteins or their downstream pathways thus represents an emerging therapeutic strategy aimed at eliminating the root of tumor regeneration [122].

As well as in the intracellular pathway, proteins within the tumor microenvironment (TME), including cytokines, chemokines, matrix metalloproteinases (MMPs), and integrins, contribute to metastasis and therapeutic resistance. MMPs such as MMP-2 and MMP-9 degrade the ECM components, facilitating tumor invasion into surrounding stroma and distant organs [123]. Integrins, transmembrane receptors for ECM proteins, mediate cell adhesion and activate focal adhesion kinase (FAK) signaling, which is essential for migration and metastasis. Certain integrin subtypes (e.g., αvβ3, α6β4) are upregulated in metastatic cancers and represent potential therapeutic targets [124].

Immune evasion is another hallmark of cancer that is mediated by protein-level changes. Tumor cells frequently overexpress immune checkpoint ligands such as PD-L1, which bind PD-1 on T cells and suppress their activity [125]. In addition, tumor-secreted enzymes like indoleamine 2,3-dioxygenase (IDO) degrade tryptophan in the TME, leading to T cell exhaustion and reduced effector function [126]. Peptides that mimic these immune-inhibitory interactions, or conversely block them, are under development as modulators of antitumor immunity [127].

Proteins also serve as clinically relevant biomarkers for cancer detection, prognosis, and therapeutic stratification. HER2, a member of the EGFR family, is frequently overexpressed in breast and gastric cancers, where it drives tumorigenesis through MAPK and PI3K signaling by forming heterodimers with other family members such as HER1/3/4. HER2 amplification correlates with aggressive disease and serves as a target for monoclonal antibodies (trastuzumab), antibody–drug conjugates, and more recently, HER2-specific peptides [128,129]. Prostate-specific antigen (PSA), although not cancer-specific, remains a widely used serum biomarker for prostate malignancies and contributes to tumorigenesis via androgen receptor and the AKT pathway modulation [59,130]. The contents described above are compiled and summarized in Table 5.

Taken together, these findings underscore the multifaceted roles that proteins and peptides play in cancer biology, not only as drivers of cell-intrinsic signaling but also as modulators of intercellular communication, immune surveillance, genomic stability, and the extracellular microenvironment. A deeper understanding of these molecular interactions provides critical insight into tumor pathophysiology and supports the development of more effective, mechanism-based therapeutic strategies.

## 4. Peptide-Based Therapeutics in Cancer

Peptide-based therapeutics have emerged as a promising and rapidly evolving modality in oncology, offering a compelling alternative to conventional small-molecule drugs and large biologics. Their distinct biochemical and structural characteristics, including high target specificity, low immunogenicity, favorable pharmacokinetic profiles, and synthetic accessibility, render them particularly well-suited for modulating intracellular signaling pathways, protein–protein interactions, and tumor-specific receptors. These properties enhance therapeutic efficacy and allow for precise molecular customization in alignment with the biological and clinical characteristics of specific cancer types [131,132].

The functional classification of anticancer peptides is typically based on their mechanisms of action [133]. One important class is the cell-penetrating peptides (CPPs), which can translocate across cell membranes to deliver therapeutic cargos such as nucleic acids, proteins, or small molecules into the intracellular compartment [134]. Representative examples include TAT and penetratin, which have also been integrated into nanoparticle-based delivery systems to improve cytoplasmic uptake [135]. Recent developments have introduced tumor-selective CPPs that respond to microenvironmental stimuli, such as acidic pH or protease activity, thereby increasing delivery precision while minimizing off-target effects [136].

A second category includes pro-apoptotic peptides, which promote programmed cell death by targeting anti-apoptotic proteins such as Bcl-2 or by mimicking endogenous death-inducing signals [102]. BH3-mimetic peptides that replicate the functional domains of BID or BIM proteins have demonstrated the ability to overcome chemotherapy resistance by facilitating mitochondrial membrane permeabilization and caspase activation [137]. Additionally, antimicrobial-like peptides such as (KLAKLAK)_2_ have been used to selectively disrupt mitochondrial membranes in tumor cells, and tumor-suppressor-derived peptides like p28, derived from p53, have shown potential in restoring defective apoptotic signaling pathways in p53-mutant cancers [138].

A third group of therapeutic peptides exerts anticancer effects by inhibiting oncogenic signaling cascades [139]. These peptides are designed to disrupt specific protein–protein interactions or receptor–ligand bindings that are critical to tumor proliferation and survival. For instance, HER2-targeting peptides have been reported to interfere with receptor dimerization and attenuate PI3K/Akt signaling, yielding antiproliferative effects in HER2-overexpressing breast cancer models [140,141]. Similar approaches have been applied to targets such as EGFR, VEGF, PD-1/PD-L1, and CXCR4, reflecting the broad versatility of inhibitory peptides in oncologic applications [142]. A summary of relevant information is provided in Table 6.

The therapeutic potential of peptides is further underscored by their favorable safety and pharmacokinetic profiles [143]. Compared to monoclonal antibodies or small-molecule kinase inhibitors, peptides generally exhibit lower systemic toxicity and reduced immunogenicity [139]. Their small size facilitates improved tumor tissue penetration and rapid clearance from healthy tissues, which is particularly advantageous in treating solid tumors with complex stromal architecture [144]. Moreover, peptides are highly amenable to chemical modification strategies such as backbone cyclization, D-amino acid substitution, N-terminal capping, and hydrocarbon stapling, which can improve their proteolytic stability, extend plasma half-life, and enhance receptor binding affinity [145].

Nonetheless, clinical application of peptides is often limited by poor metabolic stability, rapid renal clearance, and low oral bioavailability. Linear peptides are especially vulnerable to degradation by serum proteases, posing challenges in achieving sustained therapeutic concentrations in vivo [146]. To address these limitations, several advanced delivery strategies have been developed. One such approach is the use of peptide–drug conjugates (PDCs), where cytotoxic agents are covalently linked to targeting peptides via cleavable linkers [147]. These conjugates are designed to selectively accumulate in tumor tissues and release the active drug payload intracellularly. For example, camptothecin has been conjugated to RGD-motif peptides to selectively target integrin-expressing cancer cells, demonstrating effective apoptotic induction [148].

Another clinically relevant example is TH1902, a sortilin-targeting peptide–docetaxel conjugate currently undergoing evaluation in advanced solid tumors [149].

To address these limitations, several advanced delivery strategies have been developed. It is well established that linear peptides are readily cleaved by various endogenous proteases, and peptide–drug conjugate (PDC) strategies have shown promise in mitigating this limitation through multiple mechanisms [147]. The covalent attachment of cytotoxic payloads or other chemical modifications within PDCs can offer steric protection, hindering protease access to labile peptide bonds [148]. Linkers and conjugation sites can be strategically positioned to mask protease recognition sequences, thereby increasing overall metabolic stability. For example, studies have shown that conjugating drugs such as camptothecin to RGD peptides not only enhances tumor targeting but also improves resistance to proteolytic degradation, as the peptide–drug conjugate is less susceptible to enzymatic cleavage compared to the unconjugated linear peptide [149]. Furthermore, some clinically relevant PDCs, such as TH1902 (a sortilin-targeted peptide–docetaxel conjugate), have optimized linker chemistries that balance serum stability with efficient intracellular drug release, reflecting ongoing advances in protecting peptide integrity during systemic circulation [150]. While protection is not absolute, such structural modifications and conjugate designs have been proven to extend the half-life of peptide therapeutics in serum and improve their pharmacokinetic profiles [139].

In addition to conventional conjugation, stimuli-responsive peptide systems have emerged to improve intracellular targeting [151]. These include constructs that are selectively activated by reactive oxygen species (ROS), acidic pH, or tumor-associated proteases [152]. For instance, ROS-sensitive peptide–drug complexes have been designed to release their cytotoxic cargo specifically within the oxidative environment of mitochondria in cancer cells [153], thereby increasing therapeutic precision while minimizing systemic exposure.

Nanoparticle-based platforms have also played a critical role in expanding the applicability of therapeutic peptides. Encapsulation within liposomes, polymeric micelles, dendrimers, or inorganic nanoparticles can protect peptides from enzymatic degradation and prolong systemic circulation [154]. Such platforms enable co-delivery of therapeutic peptides with chemotherapeutic agents or imaging probes, enhancing both efficacy and tumor selectivity. For instance, HER2-targeting peptides have been integrated into polymeric nanoparticles or conjugated to liposomal surfaces, resulting in improved accumulation in HER2-positive tumors and enabling simultaneous delivery of multiple therapeutic agents [155].

Recent advances in synthetic biology and computational design have further accelerated peptide discovery and optimization [156]. Machine learning algorithms are increasingly used to predict peptide–receptor binding, protease susceptibility, and immunogenic potential [157]. Generative models, including transformer architectures and generative adversarial networks (GANs), are capable of proposing novel peptide sequences with optimized physicochemical and biological properties [158]. High-throughput screening platforms, such as phage and mRNA display, are now being integrated with in silico pipelines to rapidly identify high-affinity binders against diverse oncogenic targets [159]. Furthermore, the use of cell-free synthesis systems offers scalable and rapid production of peptide libraries, facilitating preclinical evaluation and lead optimization [160].

Peptides are not only being explored as monotherapies but are also under active investigation in combination with other therapeutic modalities [161]. Immunomodulatory peptides are being developed as adjuvants in cancer vaccines or as mimetics of immune checkpoint molecules, aiming to enhance antitumor immunity [162]. For example, AUNP-12, which targets the PD-1/PD-L1 axis, is designed to restore T cell activation and improve the efficacy of immunotherapy [163]. Additionally, tumor-penetrating peptides such as iRGD have been shown to improve the intratumoral delivery of chemotherapeutics and radiotherapeutics by transiently altering vascular permeability and extracellular matrix integrity [164]. Peptides derived from tumor neoantigens are also under development as personalized vaccine candidates, tailored to each patient’s unique mutational landscape and immune profile [165]. Details related to this topic are presented in Table 7.

Collectively, peptide-based therapeutics represent a highly adaptable and increasingly integral component of modern cancer therapy [166]. Their modular nature, chemical tunability, and biological specificity offer significant advantages over conventional therapeutic modalities. While challenges remain—particularly regarding metabolic stability, delivery, and manufacturing—ongoing progress in formulation technologies, computational peptide engineering, and nanotechnology is rapidly overcoming these barriers. As both academic and clinical interest in this field continues to grow, peptide therapeutics are expected to play a central role in the evolving landscape of precision oncology, particularly in the context of targeted combination regimens and personalized treatment strategies.

## 5. Protein-Based Therapeutics: Monoclonal Antibodies, Fusion Proteins, and Beyond

**Figure 2:**
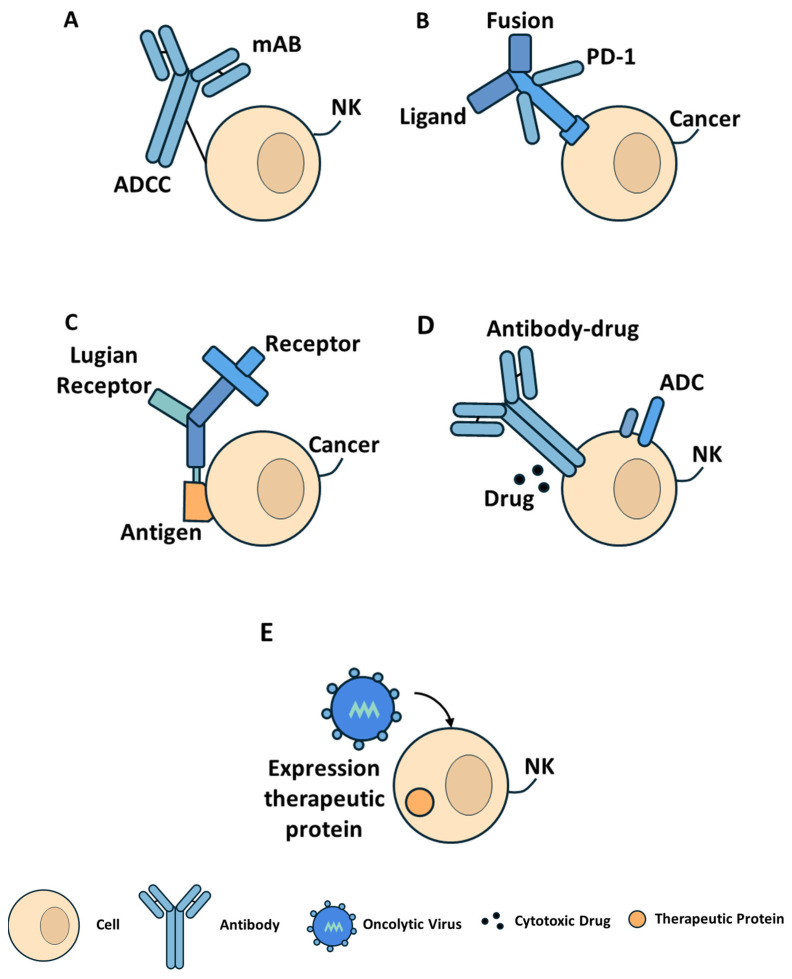
Representative mechanisms of action of major protein-based cancer therapeutics. (**A**) Monoclonal antibodies: antigen binding and immune-mediated cytotoxicity. (**B**) Immune checkpoint inhibitors: blockade of PD-1/PD-L1 or CTLA-4 to restore T cell activity. (**C**) Fusion proteins: extended half-life and immune modulation via Fc-fusion. (**D**) Antibody–drug conjugates: targeted delivery of cytotoxic payloads. (**E**) Oncolytic viruses: selective tumor lysis and induction of antitumor immunity.

Protein-based therapeutics have emerged as critical tools in the treatment of cancer, autoimmune, and infectious diseases, offering high specificity, biocompatibility, and the ability to modulate complex biological pathways. The field has evolved beyond classical monoclonal antibodies (mAbs) to encompass immune checkpoint inhibitors, engineered fusion proteins, antibody–drug conjugates (ADCs), biobetters, and novel protein scaffolds. The mechanisms underlying these diverse therapeutic modalities are illustrated in Figure 2.

Monoclonal antibodies selectively bind to specific antigens expressed on tumor or immune cells, exerting their effects through receptor blockade, ligand neutralization, antibody-dependent cellular cytotoxicity (ADCC), and complement-dependent cytotoxicity (CDC). In addition to direct cytotoxic activity, some mAbs modulate immune signaling or serve as delivery vehicles for therapeutic payloads. Trastuzumab targets the HER2 receptor and has significantly improved outcomes in HER2-positive breast and gastric cancers [167,168]. This is particularly promising given that an initial phase I clinical study demonstrated a manageable safety profile and promising activity for MM-302, a novel HER2-targeted therapy, in heavily pretreated metastatic breast cancer patients [167]. Cetuximab binds EGFR and is used in colorectal and head and neck cancers [169], while rituximab targets CD20 on B cells and is employed in non-Hodgkin lymphoma and autoimmune diseases [170,171]. These agents have spurred the development of biosimilars and next-generation antibodies with enhanced Fc-mediated functions, improved binding affinities, and greater manufacturability [172,173,174].

Immune checkpoint inhibitors restore antitumor immunity by disrupting inhibitory pathways that cancers exploit to evade T cell responses. Clinically validated targets include PD-1, PD-L1, and CTLA-4 [175,176]. Agents such as nivolumab, pembrolizumab, atezolizumab, and ipilimumab have demonstrated durable clinical responses and survival benefits in multiple malignancies, including melanoma, non-small cell lung cancer (NSCLC), renal cell carcinoma (RCC), and bladder cancer [177,178,179]. This is evidenced by the CheckMate 067 trial, where the combination of nivolumab and ipilimumab led to the longest reported median overall survival (72.1 months) in advanced melanoma. In NSCLC, the CheckMate 227 and 9LA trials confirmed survival benefits, while other studies (KEYNOTE-002, KEYNOTE-045, and CheckMate 025) highlighted that these treatments also improve or maintain patient health-related quality of life compared to chemotherapy [177,178,179]. However, limited response rates and immune-related adverse events (irAEs) remain significant challenges [179]. Current research is focused on identifying predictive biomarkers (e.g., PD-L1 expression, tumor mutational burden), exploring novel targets (e.g., TIM-3, LAG-3, TIGIT), and developing combination strategies involving chemotherapy, vaccines, or targeted agents [180,181,182].

Fusion proteins extend the functionality of biologics by combining receptor-binding domains with immunoglobulin Fc fragments, which enhances half-life, solubility, and immune effector engagement. Etanercept, a fusion of the TNF receptor with an IgG1 Fc domain, neutralizes TNF-α and is indicated for rheumatoid arthritis and other inflammatory disorders [183]. Aflibercept, composed of VEGF-binding domains fused to Fc, functions as a decoy receptor and is approved for the treatment of colorectal cancer and age-related macular degeneration [184,185]. Advances in protein engineering are facilitating the development of multifunctional fusion proteins that can simultaneously engage multiple targets [186,187].

ADCs combine the specificity of monoclonal antibodies with the potent cytotoxicity of chemotherapeutic agents via cleavable or non-cleavable linkers. T-DM1 (trastuzumab emtansine) delivers a microtubule inhibitor to HER2-positive cells [188]. In the EMILIA Phase III trial, T-DM1 significantly improved median progression-free survival (9.6 vs. 6.4 months) and overall survival (30.9 vs. 25.1 months) compared to the combination of capecitabine and lapatinib in patients with previously treated HER2-positive metastatic breast cancer [188]. Brentuximab vedotin targets CD30 and is used in Hodgkin lymphoma and anaplastic large cell lymphoma, while enfortumab vedotin binds to Nectin-4 in urothelial carcinoma [189]. Key parameters in ADC design include antigen selection, internalization efficiency, linker chemistry, drug-to-antibody ratio (DAR), and in vivo stability [190,191,192,193]. Next-generation ADCs incorporate site-specific conjugation techniques, optimized payloads such as topoisomerase inhibitors, and novel linker technologies to improve efficacy and minimize toxicity [194,195,196,197].

Biobetters are modified biologics engineered to outperform their reference products in pharmacokinetics, dosing frequency, or therapeutic efficacy. Common strategies include Fc engineering, PEGylation, and glycoengineering [198,199,200]. Examples include insulin glargine and darbepoetin alfa, which exhibit extended half-lives, and obinutuzumab, a Fc-engineered anti-CD20 antibody with enhanced ADCC and therapeutic index [201,202,203]. In parallel, alternative protein scaffolds such as nanobodies (single-domain antibodies derived from camelids), DARPins, and affibodies are being explored for their improved tumor penetration, thermal stability, and ease of production [204,205]. These platforms also offer flexibility in multi-targeting and intracellular delivery.

Oncolytic viruses represent a distinct class of protein-based therapeutics that selectively infect and lyse tumor cells while eliciting systemic antitumor immunity [206]. Some are genetically engineered to express therapeutic proteins, such as cytokines or immune checkpoint inhibitors, within the tumor microenvironment [207,208,209]. Talimogene laherparepvec (T-VEC), a modified herpes simplex virus type 1 (HSV-1) encoding GM-CSF, is approved for the treatment of advanced melanoma and functions through tumor lysis and local immune recruitment [210,211]. Clinical trials are underway to evaluate oncolytic viruses engineered to express interleukins (e.g., IL-12, IL-15) or bispecific T cell engagers (BiTEs) [209,212]. Despite ongoing challenges such as delivery efficiency, tumor penetration, and immune clearance, combination approaches with immune checkpoint inhibitors, radiotherapy, or systemic biologics offer promising avenues for enhanced therapeutic synergy [209,213]. A comparative summary of these major classes of protein-based therapeutics is provided in Table 8.

## 6. Computational Tools for Anticancer Peptide Prediction

The emergence of computational biology has significantly accelerated the discovery and optimization of anticancer peptides (ACPs) [214]. Traditional approaches relying solely on experimental screening are often limited by cost, time, and scalability [215]. To overcome these challenges, numerous in silico prediction tools and machine learning-based algorithms have been developed to identify, classify, and design ACPs with higher efficiency.

Databases such as Cancer PPD and APD (Antimicrobial Peptide Database) provide curated collections of experimentally validated ACPs, serving as essential resources for model training and benchmarking [216]. On top of these, machine learning classifiers—including support vector machines (SVM), random forests, and deep learning frameworks—have been implemented in publicly available platforms. Examples include AntiCP, iACP, and MLACP, which utilize peptide sequence features such as amino acid composition, physicochemical properties, and structural motifs to predict anticancer potential [217]. More recently, advanced deep learning architectures (e.g., convolutional neural networks, recurrent neural networks, and transformers) have enabled end-to-end feature extraction directly from raw sequences, achieving improved predictive accuracy and generalizability.

Beyond prediction, computational approaches are increasingly being integrated into de novo peptide design pipelines, where generative models and evolutionary algorithms propose novel peptide sequences with desired anticancer activity, stability, and reduced toxicity [218]. These in silico methods not only complement experimental strategies but also provide a rational framework for high-throughput candidate screening, thereby accelerating the translation of peptide-based therapeutics from bench to bedside.

## 7. Emerging Trends in Protein and Peptide Drug Development

Despite the growing interest in protein- and peptide-based therapeutics for cancer treatment, several persistent challenges continue to limit their clinical translation and long-term efficacy. These challenges span biological, technological, and regulatory domains. In this section, we discuss key barriers and explore emerging strategies that may guide future research and application.

### 7.1. Targeting Specificity and Resistance

Achieving tumor-specific delivery remains one of the central obstacles in peptide and protein therapeutics [219]. Although many cancer-associated receptors serve as targets, their basal expression in healthy tissues frequently leads to off-target toxicity and limits therapeutic selectivity [220]. Furthermore, the dynamic and heterogeneous nature of the tumor microenvironment fosters resistance through diverse mechanisms, including receptor downregulation, apoptosis evasion, and adaptive signaling alterations [24,221]. To address these limitations, targeted delivery strategies have evolved significantly. Tumor-penetrating peptides and antibody–drug conjugates (ADCs) offer improved tumor selectivity, while multifunctional nanocarriers facilitate enhanced payload delivery and controlled release [222,223].

### 7.2. Stability and Bioavailability

The physicochemical instability of peptide- and protein-based drugs remains a major barrier to clinical success [224]. Their susceptibility to enzymatic degradation—rapid for short peptides due to ubiquitous peptidases, and more pathway-specific for proteins once partially unfolded—together with poor membrane permeability, leads to rapid clearance and limited systemic bioavailability [225,226]. Several structural modifications have been explored to improve in vivo performance, including backbone cyclization, PEGylation, and incorporation of non-natural amino acids [227]. In parallel, nanocarrier systems such as liposomes and polymeric nanoparticles have demonstrated considerable potential in shielding bioactive agents from degradation while enabling controlled, site-specific release [228]. Stimuli-responsive delivery systems that respond to pH, redox state, or enzymatic activity are also under development to further refine release kinetics and tumor targeting [229,230].

### 7.3. Immunogenicity and Manufacturing Challenges

Immunogenicity represents a significant concern in protein-based therapeutics. The development of anti-drug antibodies (ADAs), often triggered by protein aggregates or impurities, can compromise efficacy and safety [231,232]. Formulation components and patient-specific immune responses further complicate this issue [225]. Although PEGylation has been employed to mask immunogenic epitopes, alternative strategies such as immune-tolerant nanocarriers are gaining traction [229,233]. From a manufacturing standpoint, ensuring consistent protein folding and bioactivity presents technical hurdles. Variability in expression systems and purification protocols can lead to product heterogeneity and reduced functionality [234,235]. Recent advances in synthetic biology and polymer–protein hybrid systems offer promising avenues for scalable and robust production pipelines [230].

### 7.4. Biomarker Reliability and Clinical Translation

The identification and application of reliable biomarkers remain a bottleneck in personalized cancer therapy. While biomarkers such as circulating tumor DNA (ctDNA) hold great potential for non-invasive diagnostics, their sensitivity is limited by tumor heterogeneity and low abundance in early-stage cancers [236,237]. In addition, the lack of standardized protocols for biomarker discovery and validation affects reproducibility across studies [77]. Operational challenges, including long turnaround times for omics-based analyses and regulatory discrepancies across regions, further hinder clinical integration [238,239]. Inadequate data sharing and poor reporting practices also reduce the translational value of promising candidates [240]. Addressing these issues will require coordinated efforts to standardize pipelines, increase data transparency, and promote cross-institutional collaboration.

### 7.5. Toward Adaptive and Personalized Therapeutics

Advancements in molecular profiling have paved the way for personalized oncology. By stratifying patients based on genetic and molecular markers, treatment efficacy can be optimized while minimizing off-target effects [241]. Technologies such as next-generation sequencing (NGS), liquid biopsy, and single-cell RNA sequencing provide dynamic insights into tumor evolution and heterogeneity [242,243]. Integrating multi-omics data enhances our understanding of cancer biology and facilitates the development of individualized therapeutic regimens [244]. Synthetic biology approaches, including custom-designed peptide vaccines and cell-based therapies, offer new levels of adaptability [245,246]. Computational modeling and predictive algorithms are increasingly employed to match molecular profiles with optimal treatment strategies [247].

### 7.6. Artificial Intelligence in Drug Design and Clinical Practice

Artificial intelligence (AI) transforms both the design and application of protein-based therapeutics. Structure prediction tools such as AlphaFold2 have dramatically accelerated the modeling of protein conformations, facilitating rapid drug design [248]. Machine learning algorithms, including convolutional neural networks and support vector machines, have demonstrated high accuracy in identifying and optimizing anticancer peptides [249,250]. Beyond discovery, AI-driven tools are applied to predict ligand binding, screen therapeutic candidates, and analyze digital pathology images for diagnostic and prognostic purposes [250,251]. In the clinical setting, AI assists in patient stratification, trial design, and real-time monitoring [252]. Nonetheless, challenges such as algorithm transparency, data security, and training bias remain critical concerns that must be addressed for broader clinical adoption [253,254].

## 8. Conclusions

Proteins and peptides have firmly positioned themselves at the forefront of cancer research and clinical practice. Their involvement in key molecular pathways that drive tumor initiation, progression, and resistance to therapy underscores their value as both biomarkers and therapeutic agents. Recent technological advancements have facilitated the precise identification and quantification of these biomolecules, while also enabling their use in increasingly sophisticated therapeutic formulations. The convergence of proteomics with other omics platforms, as well as the integration of artificial intelligence and synthetic biology, is redefining the landscape of precision medicine. These innovations are not only improving diagnostic accuracy and therapeutic efficacy but also paving the way for personalized treatment strategies tailored to individual patients. As challenges related to delivery, stability, and immunogenicity continue to be addressed, proteins and peptides are expected to remain central to future efforts in understanding and combating cancer.

## Figures and Tables

**Table 1:** Representative protein-based cancer biomarkers.

Biomarker	Cancer Type	Clinical Application	Limitations
HER2 [47,55]	Breast, gastric	Prognostic/predictive marker; guides trastuzumab therapy	Resistance in some patients
ER/PR [56]	Breast	Prognostic; endocrine therapy guidance	PR loss linked to resistance
PSA [55,57]	Prostate	Early detection and monitoring	Elevated in benign conditions
CA125/HE4 [55,58]	Ovarian	Monitor response and recurrence	Low early sensitivity, elevated in benign states
CEA [50,55]	Colorectal, GI, breast, liver, pancreatic	Prognosis, recurrence monitoring	Low specificity, expressed in non-tumor tissues
AFP [46,55]	Hepatocellular carcinoma, germ cell tumors	Diagnosis and prognosis	Elevated in hepatitis, cirrhosis
CA19-9/CA72-4 [55]	Pancreatic, gastric, colorectal	Diagnosis and monitoring	Low sensitivity, false positives in cholestasis
p53 [55,59]	Lung, colorectal, pancreatic	Predictive marker; chemotherapy resistance	Mutations reduce therapy response
LDH [60]	Solid tumors	Prognostic marker; reflects tumor burden	Non-cancer elevations
NSE [60]	Small cell lung cancer	Staging, relapse prediction	Low specificity

**Table 2:** Representative peptide-based cancer biomarkers.

Peptide Biomarker	Cancer Type	Clinical Application	Limitations
Pro-BNP [54]	Cardio-oncology	Monitor chemotherapy-related cardiac dysfunction	Not cancer-specific
Hepcidin [55]	Hepatocellular carcinoma	Iron metabolism regulation	Confounded by inflammation
Chromogranin A [56]	Neuroendocrine tumors	Diagnosis and monitoring	False positives in renal failure
Thymosin β4 [57]	Breast, colon	Linked to invasion and metastasis	Limited clinical validation
GRP/Pro-GRP [60]	Small cell lung cancer	Detection in combination with NSE	Short half-life; requires precursor stability
Micropeptides [61]	Breast cancer subtypes	Subtype-specific diagnostic/therapeutic potential	Still experimental, limited clinical translation
Tumor-homing peptides (e.g., iRGD, Angiopep2, PL3) [62]	Gastric, PDAC, prostate (depending on target)	Tumor-targeted imaging and drug delivery	Preclinical stage; limited validation in patients

**Table 3:** Representative case studies of omics-based approaches for biomarker discovery.

Cancer Type	Biomarker(s)	Omics Approach	Detection/Quantification Method
Breast cancer (HER2-positive subtype)	GRB7, INPP4B, MLPH	Integrative analysis of TCGA (transcriptomics) and CPTAC (proteomics)	RNA sequencing for transcriptomic profiling and LC–MS/MS-based quantitative proteomics for protein validation [68,69]
Ovarian cancer	WFDC2 (HE4), SLPI	Multi-omics analysis of tumor tissue and plasma (transcriptomics + proteomics)	RNA-seq for transcriptomic profiling, LC–MS/MS for proteomic validation, and ELISA for plasma protein quantification [70,71]

**Table 4:** Major quantitative techniques for protein and peptide biomarker analysis.

Technique	Detection Principle	Key Advantages	Typical Technical Limitations	Representative Examples	Potential Clinical Limitations
ELISA	Enzyme-linked antigen–antibody interaction	Clinically validated; high sensitivity [79]	Limited to single-analyte; dependent on antibody quality	Quantification of HER2 and CEA in clinical diagnostics [79,80]	Cross-reactivity; low scalability for multiplexing
MS (MRM/SRM)	Targeted mass spectrometry of specific peptides [81]	Multiplex capability; high specificity [82]	Requires advanced instrumentation and technical expertise	GP73, AFP, and DKK1 panels for hepatocellular carcinoma plasma [82,83]	Limited availability in routine clinical laboratories; need for standardization
PEA	DNA-tagged antibody proximity extension and qPCR	Ultra-sensitive; minimal sample volume; multiplexing [84]	Proprietary reagents; restricted accessibility	92-protein plasma panel for ovarian cancer diagnosis [84,85]	High cost and dependence on proprietary kits may limit adoption
CyTOF	Metal isotope-labeled antibodies with mass cytometry	Single-cell resolution; high-dimensional profiling [86]	High cost; lower throughput than flow cytometry	Immune cell subset profiling in tumor microenvironment; prediction of response to checkpoint inhibitors [86]	Complex data analysis; limited feasibility for routine clinical use

**Table 5:** Tumor-related proteins and their roles in cancer progression.

Protein/Peptide		Role	Associated Pathways and Functions
Protein	Bcl-2	Inhibitor of apoptosis	Suppresses mitochondrial apoptosis and enhances cell survival by interacting with proteins like Beclin-1.
	p53	Genomic gatekeeper	Senses DNA damage and activates genes for cell cycle arrest, DNA repair, and apoptosis. Mutations lead to a loss of these protective functions.
	ATR, ATM, CHK1/2, BRCA1/2	Regulators of the DNA damage response (DDR)	Control DNA repair processes. Their mutation or inactivation can lead to genomic instability.
	EGF, VEGF, TGF-β	Cell signaling molecules	EGF and VEGF promote cell growth and angiogenesis. TGF-β acts as a tumor suppressor in early stages but promotes tumor progression in later stages.
	EGFR, VEGFR-2, HER2	Receptor tyrosine kinases	EGFR and HER2 activate the MAPK and PI3K pathways. VEGFR-2 promotes the proliferation of vascular endothelial cells.
	Nanog, Sox2, Oct4	Cancer stem cell (CSC) maintainers	Sustain the self-renewal and tumor-initiating capacity of CSCs, contributing to tumor recurrence.
	MMP-2, MMP-9, Integrins	Promoters of metastasis	MMPs degrade the extracellular matrix (ECM) to facilitate invasion, while Integrins mediate cell adhesion and migration to promote metastasis.
	PD-L1, IDO	Mediators of immune evasion	PD-L1 suppresses T-cell activity to help cancer cells evade immune attacks. IDO impairs the function of immune cells.
	PSA	Prostate cancer biomarker	A serum biomarker for prostate cancer that also modulates the androgen receptor and AKT pathways.
Peptide	Emerging peptide-based approaches	Therapeutic modulators	Mimic or inhibit DDR proteins to make cancer cells more sensitive to DNA-damaging agents.
	HER2-specific peptides	Targeted therapeutics	Used as a therapeutic strategy to target HER2-positive tumors.
	Immune-inhibitory peptides	Immunotherapeutics	Used to block immune-inhibitory interactions to modulate anti-tumor immunity.

**Table 6:** Therapeutic peptide classes and their mechanisms of action.

Peptide Type	Primary Function	Representative Mechanism	Examples
Cell-penetrating peptides (CPPs)	Intracellular delivery of therapeutic payloads	Cross plasma membranes and transport small molecules, proteins, or nucleic acids	TAT [135], penetratin [134]
Pro-apoptotic peptides	Induction of programmed cell death	Target anti-apoptotic proteins (e.g., Bcl-2), promote mitochondrial membrane permeabilization	BH3-mimetic peptides [137]
Inhibitory peptides	Interruption of oncogenic signaling	Disrupt ligand-receptor interactions or downstream signaling pathways	HER2-blocking peptides [140,141]

**Table 7:** Challenges and strategies in peptide therapeutics.

Limitation	Solution	Example/Technology
Proteolytic degradation	Structural modification	Cyclization, D-amino acid substitution, N-terminal capping [146,147]
Poor bioavailability	Alternative delivery systems	Nanoparticles (liposomes, dendrimers), PEGylation [152]
Short plasma half-life	Enhanced pharmacokinetics	Backbone stabilization, conjugation with long-circulating carriers [148]
Lack of tumor specificity	Targeted delivery platforms	Peptide–drug conjugates (PDCs), tumor-penetrating peptides [164,165]

**Table 8:** Comparison of major classes of protein-based therapeutics. Monoclonal antibodies are presented with subcategories, including conventional anti-cancer antibodies and immune checkpoint inhibitors.

Therapeutic Class	Representative Drugs	Mechanism of Action	Indications	Advantages	Limitations	Refs.
Monoclonal antibodies (anti-cancer targets)	-Trastuzumab -Cetuximab	Antigen-specific binding, ADCC/CDC activation	Breast cancer, CRC	High specificity, stability	Drug resistance, limited tissue penetration	[167,168,169]
Monoclonal antibodies (immune checkpoint inhibitors)	-Nivolumab -Pembrolizumab	Blocking T cell inhibitory pathways (PD-1/PD-L1, CTLA-4)	Melanoma, NSCLC	Long-term survival, broad tumor scope	Limited responders, immune-related AEs	[175,176,177,178,179]
Fusion proteins	-Etanercept -Aflibercept	Ligand trapping, Fc fusion to extend half-life	Autoimmune diseases, anti-angiogenesis	Improved in vivo stability	Potential immunogenicity	[183,184,185]
Antibody–drug conjugates (ADCs)	-T-DM1 -Brentuximab vedotin	Antibody-targeted cytotoxic drug delivery	HER2+ breast cancer, etc.	High precision therapy	Linker stability, toxicity concerns	[188,189,190,191,192,193]
Biobetters	-Obinutuzumab -Darbepoetin alfa	Enhanced pharmacokinetics via structural modifications	Lymphoma, anemia	Reduced dosing frequency, improved efficacy	High development cost, patent issues	[198,199,200,201,202,203]

## Data Availability

Data sharing is not applicable.
